# Nitrogen Functionalization of CVD Grown Three-Dimensional Graphene Foam for Hydrogen Evolution Reactions in Alkaline Media

**DOI:** 10.3390/ma14174952

**Published:** 2021-08-30

**Authors:** Daniela Ion-Ebrașu, Radu Dorin Andrei, Stanică Enache, Simona Căprărescu, Constantin Cătălin Negrilă, Cătălin Jianu, Adrian Enache, Iulian Boerașu, Elena Carcadea, Mihai Varlam, Bogdan Ștefan Vasile, Jianwei Ren

**Affiliations:** 1 National Institute for Cryogenics and Isotopic Technologies ICSI-Rm. Valcea, ICSI Energy, Uzinei Street, No. 4, 240050 Ramnicu Valcea, Romania; daniela.ebrasu@icsi.ro (D.I.-E.); radu.andrei@icsi.ro (R.D.A.); stanica.enache@icsi.ro (S.E.); catalin.jianu@icsi.ro (C.J.); Adrian.enache@icsi.ro (A.E.); elena.carcadea@icsi.ro (E.C.); mihai.varlam@icsi.ro (M.V.); 2 Inorganic Chemistry, Physical Chemistry and Electrochemistry Department, Faculty of Applied Chemistry and Materials Science, University POLITEHNICA of Bucharest, Gh. Polizu Street, No. 1–7, 011061 Bucharest, Romania; 3 National Institute for Materials Physics, P.O. Box MG-7, 77125 Bucharest, Romania; catalin.negrila@infim.ro; 4 National Research Center for Micro and Nanomaterials, Faculty of Applied Chemistry and Materials Science, University POLITEHNICA of Bucharest, Splaiul Independentei Street, No. 313, 060042 Bucharest, Romania; iulianboerasu@gmail.com (I.B.); bogdan.vasile@upb.ro (B.Ș.V.); 5 Department of Mechanical Engineering Science, University of Johannesburg, Cnr Kingsway and University Roads, Auckland Park, Johannesburg 2092, South Africa; jren@uj.ac.za

**Keywords:** graphene, chemical vapor deposition, nitrogen-doping, hydrogen evolution reaction

## Abstract

Three-dimensional graphene foam (3D-GrFoam) is a highly porous structure and sustained lattice formed by graphene layers with sp^2^ and sp^3^ hybridized carbon. In this work, chemical vapor deposition (CVD)—grown 3D-GrFoam was nitrogen-doped and platinum functionalized using hydrothermal treatment with different reducing agents (i.e., urea, hydrazine, ammonia, and dihydrogen hexachloroplatinate (IV) hydrate, respectively). X-ray photoelectron spectroscopy (XPS) survey showed that the most electrochemically active nitrogen-doped sample (GrFoam3N) contained 1.8 at % of N, and it exhibited a 172 mV dec^−1^ Tafel plot associated with the Volmer–Heyrovsky hydrogen evolution (HER) mechanism in 0.1 M KOH. By the hydrothermal process, 0.2 at % of platinum was anchored to the graphene foam surface, and the resultant sample of GrFoamPt yielded a value of 80 mV dec^−1^ Tafel associated with the Volmer–Tafel HER mechanism. Furthermore, Raman and infrared spectroscopy analysis, as well as scanning electron microscopy (SEM) were carried out to understand the structure of the samples.

## 1. Introduction

With the rapid development of the world economy, the current energy system has posed challenges to the environment and the sustainability of energy resources. With the ongoing efforts to transition energy systems, the defining of a new carrier of energy that completely removes carbon from this chain is vital to avoiding the major problems facing the global economy. The transition to clean energy cannot be conceived without involving, to a certain extent, hydrogen, i.e., H_2_ gas produced by renewable energies.

Nowadays, electrolysis is considered one of the most favorable solutions to producing hydrogen for transport and mobile applications to alleviate the depletion of fossil fuels and maximize the utilization of renewable energy [1,2]. Compared to the catalytic steam reforming of fossil fuel, water electrolysis is considered to be the most flexible and sustainable long-term renewable energy storage solution at different scales. Typically, it utilizes extra-generated renewable electricity to produce hydrogen, and then converts it back into electricity on demand by using different devices such as fuel cells.

Depending on the electrolyte that is used in the system, water electrolysis can take place in alkaline, acidic and solid oxide electrolytes. Alkaline electrolytes are distinguished in liquid and solid anion exchange membrane (AEM) electrolytes, while a solid proton exchange membrane (PEM) is used as acidic ionomer. During electrolysis, a voltage higher than minimum 1.2 V is necessary to split water into hydrogen (HER) at the cathode and oxygen (OER) at the anode [3]. In alkaline electrolysis, the active ion is hydroxide (OH^−^), which is formed after the electrochemical decomposition of the alkaline solution. Hydroxide ions are oxidized at the anode forming oxygen gas, and water is reduced at the anode generating hydrogen. During electrochemical water reduction, two electrons are necessary to produce hydrogen gas at the cathode [4,5].

HER is slower in alkaline electrolytes than that in acid [6]. To date, the most studied catalyst is platinum supported on carbon black (Pt/C). According to other studies reported in the literature [7,8], the Tafel slope value of the commercially Pt/C catalyst measured in 0.5 M H_2_SO_4_ is 30 mV dec^−1^ and between 55 and 60 mV dec^−1^ in 1 M KOH, respectively. Nevertheless, the limited resources of Pt-based catalysts and their high costs are considered prohibitive, therefore scientists are looking for innovative non-noble metal materials with high efficiency and low cost for electrochemical water splitting to produce hydrogen [9].

To overcome these obstacles, one feasible idea is graphene functionalization with metal-free catalysts (e.g., N, B, S, Cl, F, P, Br, or I), with high electrochemical activity and stability to alkaline media, adjustable structural composition, and low cost [10,11]. Graphene functionalization is likely achieved by creating covalent bonds between the dopants and graphene substrate, which change the graphene local sp^2^ to sp^3^ hybridization, create defects, and decrease electronic conductivity [12]. Incorporating nitrogen atoms into the graphene structure is considered a route to creating a valance band and, consequently, to change its electronic structure [13]. Thus, it is possible to tune the electrochemical properties of nitrogen-doped graphene to synthetize a stable and highly efficient non-metallic catalyst for oxygen reduction (ORR) [14], hydrogen production (HER) [15,16], and oxygen production (OER) reactions [17]. Li et al. [15] indicated that the free-standing N-enriched carbon foam@WS_2_ nanoflakes prepared by a thermal evaporation process, can be used as the working electrodes for HER in water splitting. These materials exhibited an excellent electrocatalytic activity, favorable electrochemical stability, and durability.

Nitrogen graphene functionalization can be carried out in situ by assisted chemical vapor deposition (CVD) using different nitrogen sources (e.g., ammonia, pyridine, poly-pyrrole, and acetonitrile) [18], and ex situ using thermal annealing [19], pyrolysis [20], arc-discharge [21], hydrothermal [14,22,23,24] and solvothermal [25] methods, plasma [26] and microwave [27,28] treatment, wet chemical synthesis [29,30], and lyophilization-assisted heat treatment [31]. Deng et al. [25] performed the synthesis of N-doped graphene by the solvothermal method. They suggested that increasing the amount of nitrogen doping can significantly increase the electron density.

In nitrogen-doped graphene, there are several possible positions in which nitrogen can substitute carbon, such as pyridinic N and graphitic N/quaternary, that correspond to the sp^2^ hybridization, pyrrolic N with sp^3^ hybridization, amines, and N-oxides of pyridinic–N [26,32]. In the case of the pyridinic N and pyrrolic N configuration, the nitrogen-doped graphene is less conductive than pristine graphene, while graphitic N increases the graphene’s electrical properties [33,34,35].

The present study investigates the hydrothermal nitrogen functionalization of CVD-grown three-dimensional graphene foam (3D-GrFoam) using three concentrations of urea together with hydrazine and ammonia as doping elements for HER in alkaline environments. Moreover, the hydrothermal method was used to embody platinum within the pristine and nitrogen-functionalized graphene foam. This approach is thought to produce low-cost and reproducible electrodes, with large areas (up to semi-industrial dimensions of 225 cm^2^) and increased electrocatalytic activity for HER. The materials developed within the work may play the double role of a porous flow field replacing the metal foam and catalyst support. As consequence, mass transport (water and gases) through the graphene pores will be enhanced, and the weight of the device and corrosion in an alkaline electrolyte may decrease. Morphological, structural and compositional analysis was performed by scanning electron microscopy (SEM), X-ray photoelectron spectroscopy (XPS), Raman spectroscopy, and infrared spectroscopy with Fourier transformation (FTIR). Electrical conductivity at room temperature (RT) was determined by a two-point house-made conductivity cell. HER was studied by linear sweep voltammetry and cycling voltammetry in an alkaline environment, using a three-electrode electrochemical cell and a rotating disk electrode (RDE).

## 2. Materials and Methods

### 2.1. Materials and Reagents

Nickel foam from Alfa Aesar (Thermo Fisher (Kandel) GmbH, Kandel, Germany) with 1.6 mm thickness and 70% porosity was used as received as the catalytic substrate for graphene growth. Ethylene (C_2_H_4_, 99.99%) and argon (99.999%) from Messer (Messer Schweiz AG, Seonerstrasse, Lenzburg, Switzerland), and hydrogen (99.999%) produced onsite by electrolysis were used during the CVD process. Hydrochloric acid (36.5–38.0%) and demineralized water (DW) (18.2 MΩ cm) produced on-site were employed for nickel foam substrate removal. For the graphene nitrogen functionalization step, urea, ammonium hydroxide solution (28–30%) and hydrated hydrazine (50–60%) from Sigma Aldrich (Merck Romania SRL, an affiliate of Merck KGaA, Darmstadt, Germany) were used. The materials used for Pt/GrFoam synthesis were dihydrogen hexachloroplatinate (IV) hydrate (H_2_PtCl_6•x_H_2_O, 99.999%, Alfa Aesar, Thermo Fisher (Kandel) GmbH, Kandel, Germany), ethylene glycol (99%), and sodium hydroxide (Merck KGaA, Darmstadt, Germany). Pt black nominally 40% carbon (Alfa Aesar, Thermo Fisher (Kandel) GmbH, Kandel, Germany), ethanol (Merck Romania SRL, an affiliate of Merck KGaA, Darmstadt, Germany) and 5 wt % Nafion solution were used to prepare the Pt/C solution.

### 2.2. Three-Dimensional Graphene Foam Growth by Chemical Vapor Deposition (CVD) Method

The three-dimensional graphene foam (3D-GrFoam) samples were grown by the CVD method using the EasyTube 3000ECT CVD installation/CVD Equipment Corporation installation (CVD Equipment Corporation, Central Islip, NY, USA). The detailed experimental conditions are presented elsewhere [35]. Typically, 200 standard cubic centimeters per minute (sccm) of an ethylene (C_2_H_4_) carbon source was cracked under atmospheric pressure at 1000 °C, in the presence of argon and hydrogen. The growth process was preceded by nickel substrate annealing in hydrogen atmosphere for metal template cleaning and activation. The transition of 3D-GrFoam was performed by 20 h of boiling at 90 °C in 3 M hydrochloric acid, until the acid solution turned clear. The samples were washed with DW and dried at room temperature (24 ± 1 °C). This procedure obtained free-standing 3D-GrFoam with a 1.6 mm average thickness and 12 mg cm^−3^ mass density, equivalent to a 25 cm^2^ sample surface.

### 2.3. Three-Dimensional Graphene Foam Nitrogen Functionalization

**Figure 1:**
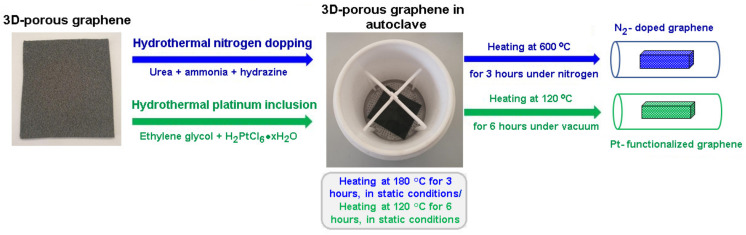
Scheme of the hydrothermal nitrogen doping and platinum functionalization of the 3D-porous graphene foam.

The nitrogen functionalization of three-dimensional (3D)-porous graphene was conducted using the hydrothermal method. The experiments were carried out in a 1000 mL Teflon HYD-1000 autoclave from Tefic Biotech (dft technology GmbH, Postfach, Neumünster, Germany). Three different urea concentrations were used to obtain different (3D)-porous graphenes: 0.2135 g (GrFoam1N), 0.4144 g (GrFoam2N), and 0.6405 g (GrFoam3N), respectively, that were dissolved in 80 mL DW. The urea solution was stirred for 5 min, while ammonia was dropped until the pH reached 10. Subsequently, 2 mL of hydrated hydrazine was added as reducing agent, and afterwards the solution was introduced into the autoclave [14]. In order to prevent the graphene foam from breaking, a special Teflon support was designed for its slow diving and supporting during the hydrothermal process. The next step was to keep the autoclave in an oven at 180 °C for 3 h, in static conditions. In order to stabilize the nitrogen within the graphene matrix, after completely cooling, the samples were introduced in a nitrogen-controlled oven and kept for 1 h at 200 °C, and then for 3 h at 600 °C (Figure 1). The inclusion of Pt into the graphene foam matrix was carried out in the same autoclave by soaking 0.0358 g of graphene into 80 mL of ethylene glycol for 1 h [14]. Then, the graphene foam was removed from the Teflon vessel, and a solution containing 10.36 mg of dihydrogen hexachloroplatinate (IV) hydrate and 3 mL DW was added gradually. The pH of the mixture was adjusted to 11 by adding 2.5 M sodium hydroxide. Next, graphene foam was introduced into the solution and treated for 6 h at 120 °C. Lastly, the Pt-embedded graphene foam (GrFoamPt) was washed with DW and dried for 6 h at 80 °C under vacuum (Figure 1). The same procedure was applied for incorporating Pt into the GrFoam1N to obtain the GrFoam1NPt sample.

## 3. Characterization of Graphene Foam

### 3.1. X-ray Photoelectron Spectroscopy (XPS)

The XPS measurements were undertaken in a high-vacuum SPECS spectrometer (SPECS Surface Nano Analysis GmbH, Voltastrasse, Berlin, Germany). The excitation source was a monochromatic XR50M source operated at 300 W, with a radiation energy of 1486.6 eV (Al Kα) and a FWHM (full width at half maximum) of 0.3 eV. The spectrometer is based on a PHOIBOS 150 hemispherical analyzer with an ultimate resolution of 0.44 eV (defined as the FWHM of a recorded Ag3d^5/2^ spectral line) and 9 channel Tron detector unit. The C1s, O1s, N1s, and Pt^4f^ spectra were recorded with a pass energy of 20 eV, and a channel energy width of 0.05 eV, while the extended spectra was recorded with a pass energy of 50 eV. The spectra fitting was processed using Voight functions and the Shirley background subtraction method in the spectral data processor software. The relative sensitivity factors provided by the spectrometer manufacturer were used.

### 3.2. Scanning Electron Microscopy (SEM)

Scanning electron microscopy (SEM) characterization was carried out by a Versa Three-Dimensional (3-D) DualBeam Field Emission scanning electron microscope (FEI Company, Hilsboro, WA, USA) under high vacuum (<3 × 10^−4^ Pa), using an accelerating voltage of 20 kV and a 5.5 spot size. The complex topography of the analyzed surfaces was obtained using a chamber Everhart–Thornley Detector (ETD) for secondary electron signal detection. A retractable circular backscatter detector (CBS) was utilized to distinguish the compositional contrast of the samples.

### 3.3. Raman Spectroscopy

Raman spectroscopy was carried out by using alpha300 RAS+ system from WITec (WITec Wissenschaftliche Instrumente und Technologie GmbH, Ulm, Germany). The system is equipped with a 75 mW Ar laser (λ = 532 nm) in conjunction with an inverted microscope (with 20× magnification) and a spectrograph (with 600 grooves per mm grating) connected to a CCD camera that records one spectrum in less than 5 msec over the spectral range between 100 and 3800 cm^−1^ and with an average spectral resolution of ±1.50 cm^−1^. In order to discern the overall Raman features from the background, the laser intensity as tuned using an external variable aperture. The acquisition time was set as high as 3 s. Since the sample topology varied from spot to spot, we decided to carry out surface scans of given areas (i.e., 25 × 25 µm^2^) comprising 25 × 25 equally displaced measurement spots. The overall spatial resolution of the focused laser beam was 0.61 × λ/NA = 0.811 µm, with λ laser wavelength and NA = 0.40 of the numerical apertures of the 20× magnification objective lens. All spectra were corrected for noise and cosmic rays, and the background contribution was subtracted using a smooth polynomial function. For spectral analysis, a set of five Lorentz distributions together with a floating baseline were used to de-convolute all Raman spectra in the range between 1200 cm^−1^ and 1700 cm^−1^. The Lorentz distributions are associated with the Raman vibration referred in literature as the D1, D, D2, G, and D’ bands. They arise from amorphous carbons (i.e., D1 and D2) and the more ordered graphene-like structures (i.e., D, G, and D’). More information on these Raman bands are found elsewhere [36,37,38].

### 3.4. Fourier Transformation Infrared Spectroscopy (FTIR)

Fourier transformation infrared spectroscopy (FTIR) spectra were recorded in transmittance in a domain of 400–4000 cm^−1^ and at 4 cm^−1^ resolution in a domain of 400–4000 cm^−1^, using a Frontier FT-NIR spectrometer from Perkin Elmer (PerkinElmer, Inc., Waltham, MA, USA).

## 4. Hydrogen Evolution Reaction (HER)

The hydrogen evolution reaction (HER) of the graphene foam-based catalysts was studied using a three-electrode cell formed by a platinum wire counter electrode, 3.0 M Ag/AgCl Metrohm reference electrode (Metrohm Analytics Romania SRL, Bucharest, Romania), and a rotating disk electrode (RDE) (RDE 710, Gamry Instruments, Louis Drive, Warminster, PA, USA). The electrochemical characterization was registered by PARSTAT 2273 potentiostat/galvanostat (Princeton Applied Research, Advanced Measurement Technology, Inc., South Illinois Avenue, Oak Ridge, TN, USA), and the potentials are reported vs. Ag/AgCl. The samples sized 0.196 cm^2^ were placed on the stainless steel tip and fixed in the demountable sample holder of an RDE from Origalys (OrigaLys ElectroChem SAS, Les Verchères 2, Rillieux-la-Pape, Lyon, France). For comparison, a Pt/C was prepared using 5 mg Pt black nominally 40% of carbon, 242 µL isopropanol, 20 µL Nafion 5 wt%, and 10 µL DW, then stirred overnight. Then, 10 mL of Pt/C aliquot was dropped on the 0.196 cm^2^ glassy carbon, equivalent to 375 µg Pt/cm^2^. Before Pt catalyst deposition, the glassy carbon was polished to mirror finish with 1.0 μm alumina and 0.05 μm diamond paste and cleaned by ultrasonication in DW [39]. Linear sweep voltammetry (LSV) was carried in a domain of 0–0.8 V with a scan rate of 0.005 V s^−1^ over a range of rotation rates (250 to 2000 rpm). All the measurements were performed at room temperature (24 ± 1 °C), in 0.1 M KOH solution that was purged for one hour with high purity nitrogen.

The electro oxidization at the anode and the electrochemical production reaction are as follows [40]:

(1) $$H_{2}O+2e^{-}\to H_{2}+2{\mathrm{OH}}^{-}\;\;(\mathrm{cathode})$$

(2) $$2{\mathrm{OH}}^{-}\to \frac{1}{2}O_{2}+H_{2}O+2e^{-}\;\left(\mathrm{anode}\right)$$

According to the Equation (2), during an electrochemical water reduction two electrons are necessary to produce hydrogen gas at the cathode. Depending on the material catalytic activity of the electrode, the HER takes place through two mechanisms, Volmer–Heyrovsky and Volmer–Tafel, as follows [41]:

(3) $$H_{2}O+M+e^{-}\to M-H_{ads}+{\mathrm{OH}}^{-}\;(\mathrm{Volmer}\;\mathrm{step}—\mathrm{water}\;\mathrm{dissociation})$$

(4) $$H_{2}O+M-H_{ads}\;+e^{-}\to H_{2}+M^{+}+{\mathrm{OH}}^{-}\;(\mathrm{Heyrovsky}\;\mathrm{step})$$

(5) $$M-H_{ads}\;\to \;2M+H_{2}\;(\mathrm{Tafel}\;\mathrm{step})$$

In the Volmer step, the water is reduced and *M-H_ads_* intermediates are formed on the cathode. The Tafel slope is obtained from the linear sweep voltammograms measured at a specific speed of the rotating disk electrode (RDE) by plotting the electrode potential versus the logarithm of the absolute current density. The value of the Tafel plot (mV dec^−1^) reflects the reaction mechanism. If this value is small, the charge transfer between the electrolyte and the electrode is fast.

## 5. Results and Discussions

### 5.1. X-ray Photoelectron Spectroscopy (XPS)

**Figure 2:**
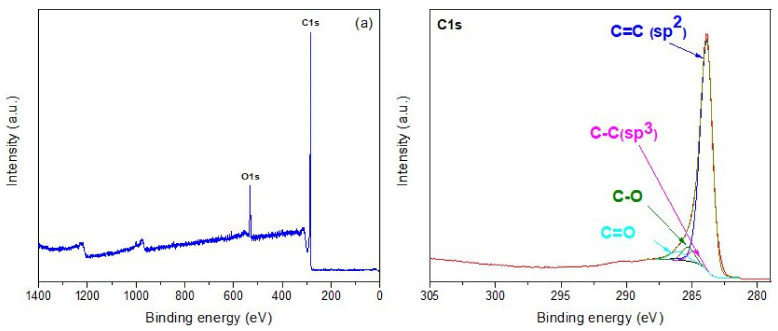

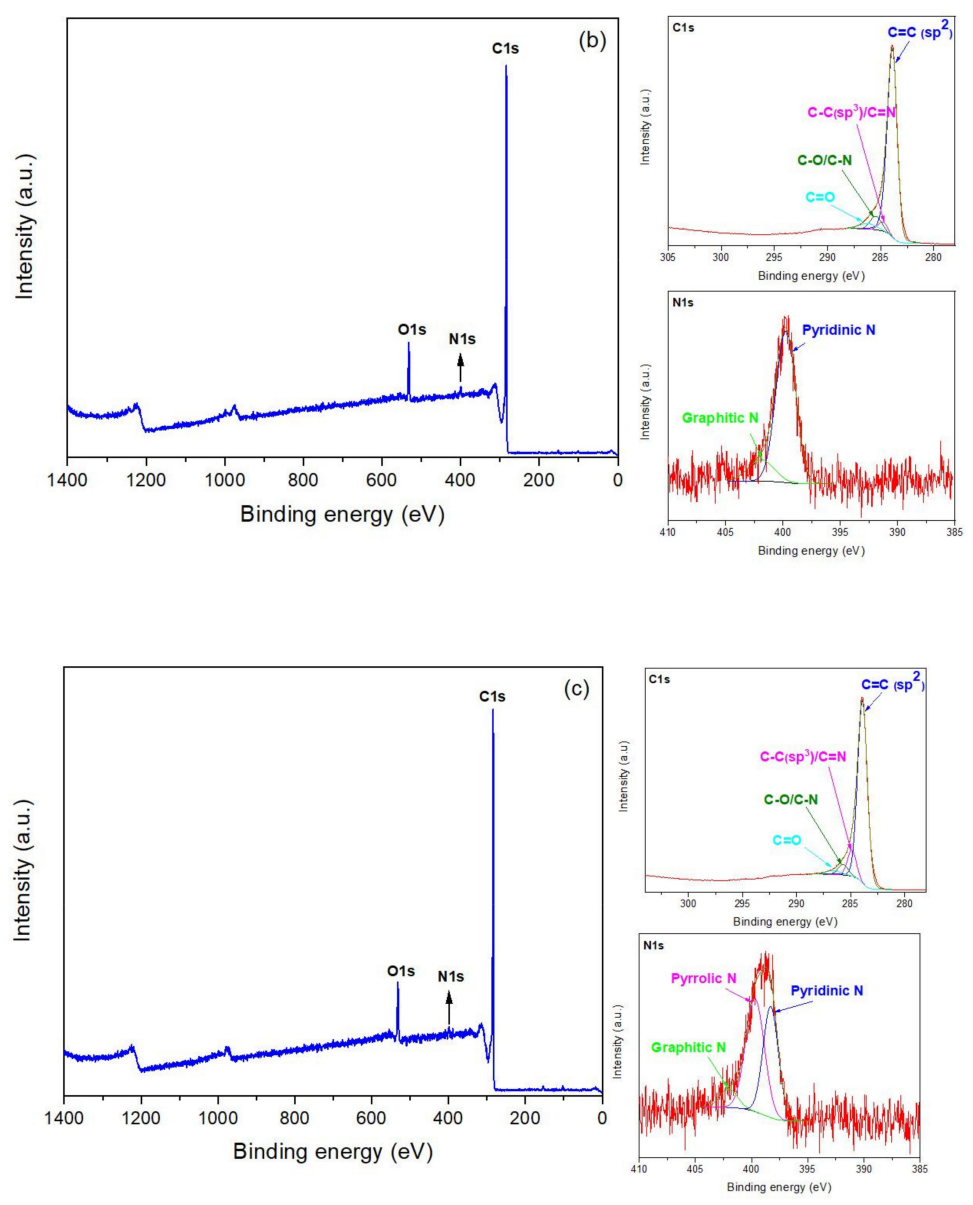

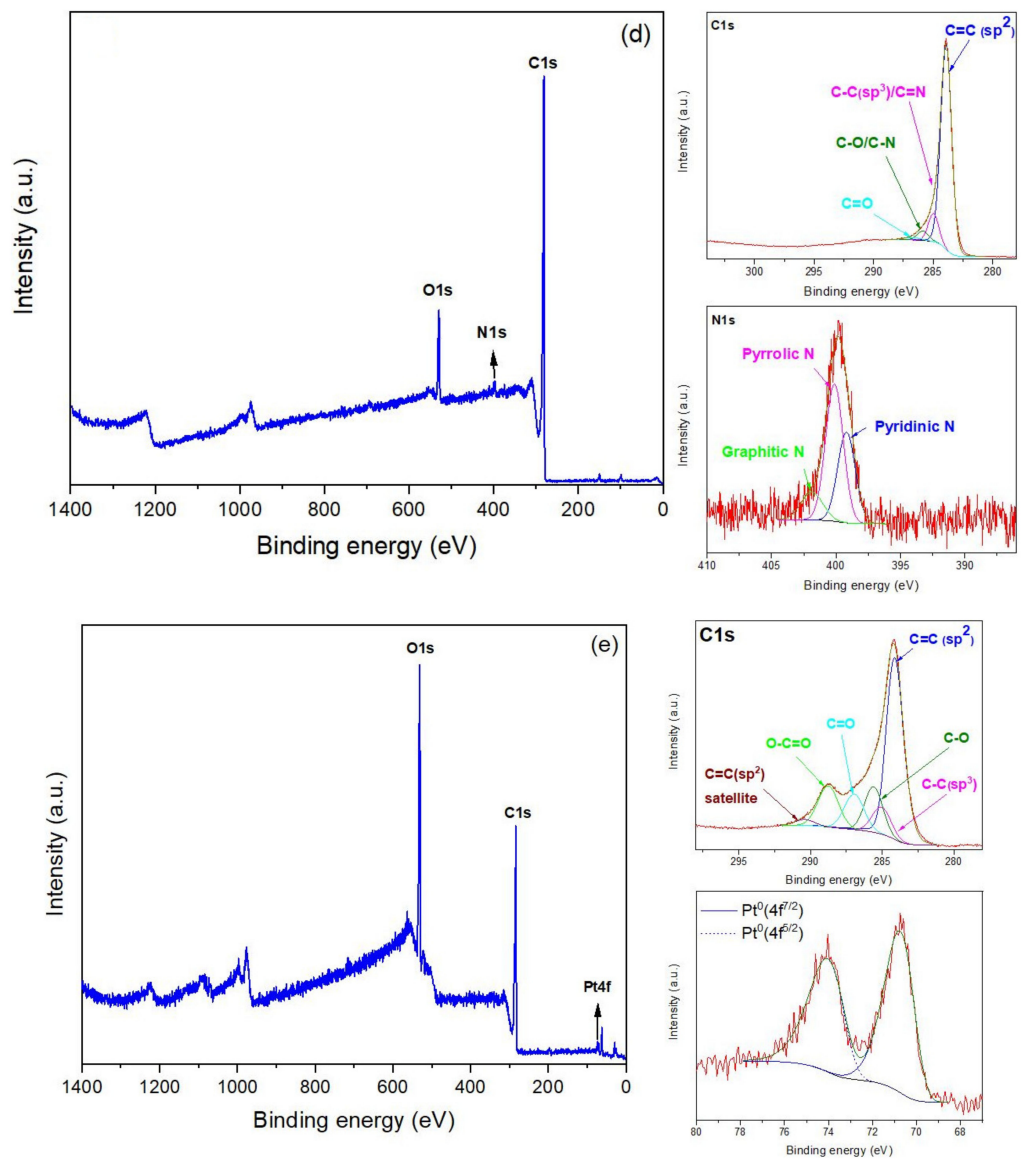

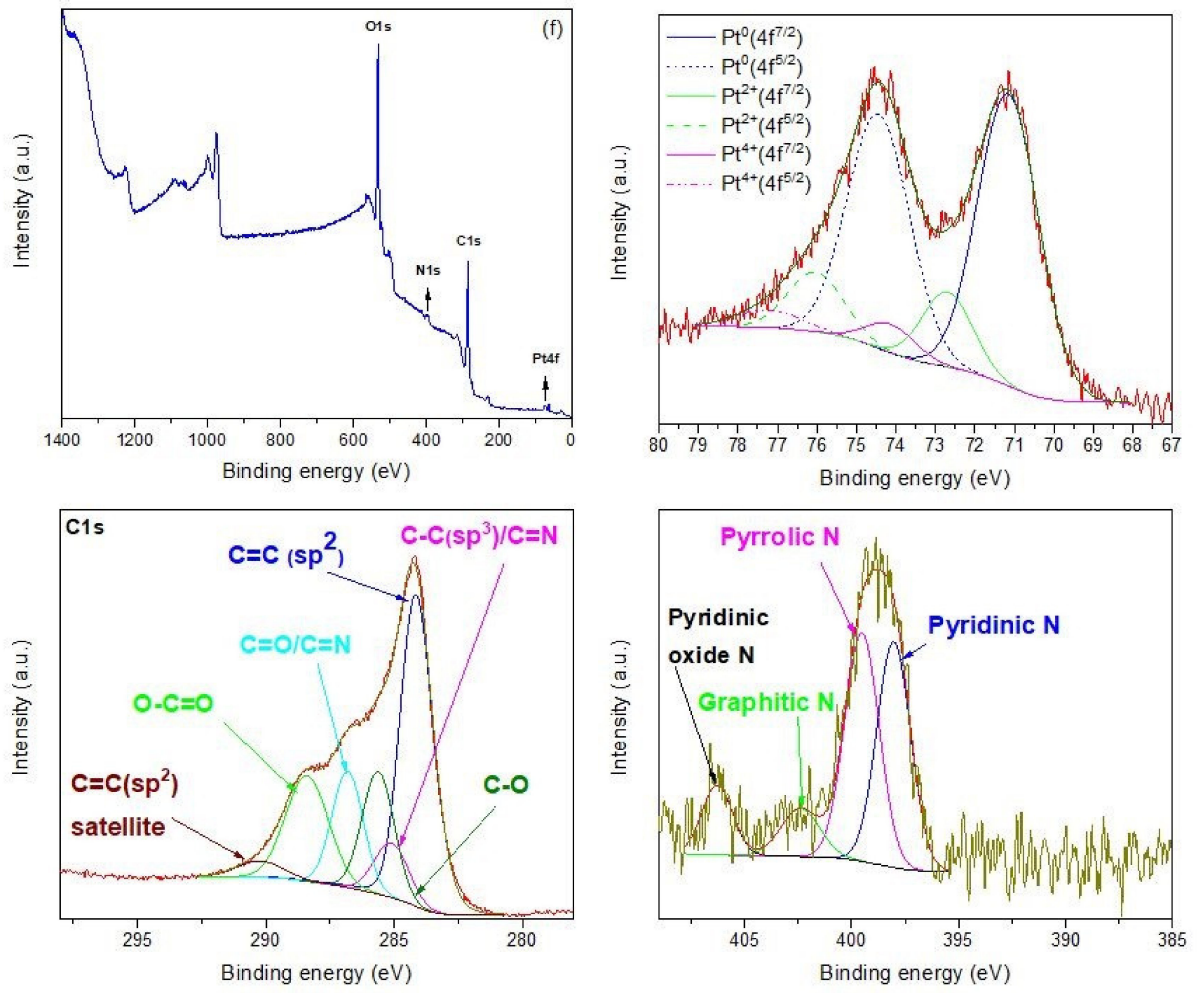
XPS surveys of (**a**) full survey spectrum of GrFoam with C1s inset; (**b**) full survey spectrum of GrFoam1N with C1s and N1s; (**c**) full survey spectrum of GrFoam2N with C1s and N1s; (**d**) full survey spectrum of GrFoam3N with C1s and N1s; (**e**) full survey spectrum of GrFoamPt with C1s and Pt^−4f^; (**f**) full survey spectrum of GrFoam1NPt with C1s inset, and N1s and Pt^−4f^.

The X-ray photoelectron spectroscopy (XPS) method was used to investigate the nitrogen doping level and platinum inclusion into the graphene foam matrix. Figure 2a–f presents the survey spectra of the pristine GrFoam, the nitrogen-doped graphene foam (GrFoam1N-GrFoam3N), and the platinum-included foam (GrFoamPt, GrFoam1NPt), respectively. All the spectra display 2 peaks associated with carbon (C1s) and oxygen (O1s). The oxygen peak presents in all the samples and is due to contamination during CVD graphene foam growth at atmospheric pressure and the aqueous HCl etching solution.

Table 1 lists the surface atomic composition for nitrogen and platinum functionalized graphene foam determined from XPS measurements.

As observed from the inset of Figure 2a, four C1 peaks are situated at 283.88 eV, 284.80 eV, 285.29 eV, and 286.05 eV, associated with C=C sp^2^ hybridization, C-C sp^3^ hybridization, C-O, and C=O, respectively. Comparing these values with the one presented in Figure 2b–f and Table S1 from the Supplementary Materials, where the peak position and concentration distribution of nitrogen and platinum of the C1s are shown, we can concluded that introduction of defects such as nitrogen and platinum shifts the binding energies to higher values because of their links with the graphene foam structure. Moreover, the C=C sp^2^ concentration decreased from 86.7 at % for GrFoam to 85.5 at %, 82.3 at % and 84 at % for GrFoam1N, GrFoam2N and GrFoam3N, respectively. The increasing C=C sp^3^ composition can be explained by nitrogen inclusion into the graphene matrix. Santhosh et al. [26] showed that nitrogen was successfully incorporated in graphene nanowalls (CNWs). They reported that the main peak of CNWs at 284.6 eV corresponds to the graphite-like sp^2^ C–C bond (graphitic-C), and that the peaks at 285.5 eV originate from the sp^3^- bonded carbon atoms. All the samples present small amounts of C-O and C=O species due to their oxidation during processing. In the case of GrFoamPt and GrFoam1NPt, the C=C sp^2^ peaks were situated at 284.13 eV and 284.15 eV, and the concentration was 53.7 at % and 43.4 at %, respectively. Furthermore, the concentration of oxidation species increased during platinum functionalization, as is shown by the O-C=O species present at 288.76 eV and 288.38 eV, having 11.2 at % and 14 at % content. The sp^2^ hybridization characteristic shake-up satellite peaks from 290.41 eV and 290.22 eV present in these two samples are due to the π–π* (HOMO–LUMO) transition formed during the substitutional platinum introduction into the graphene structure. Moreover, the position of the C1s peak commonly moves to higher binding energies after graphene nitrogen and platinum functionalization [26].

The deconvoluted XPS spectra of nitrogen presented in Figure 2b–d and Table S2 show that there are four possible nitrogen N1s type components corresponding to pyridinic N and graphitic N, which are sp^2^ hybridized, sp^3^ hybridization pyrrolic N, and pyridinic oxide N [26]. The Pyridinic N position shifted from 399.74 eV for GrFoam1N to the lower binding energy 398.30 eV, 399.18 eV and 398.03 eV, assigned to GrFoam2N, GrFoam3N, and GrFoam1NPt, respectively. The pyridinic concentration decreased progressively from 84.3 at % in GrFoam1N to 41.4 at % in GrFoam2N, 34.5 at % in GrFoam3N and 39 at % for GrFoam1NPt, being compensated by the pyrrolic N content arising and growing with the increase in the urea concentration. Even though the pyrrolic N component was not present in GrFoam1N, it was spotted in GrFoam1NPt at 399.51 eV and 40.5 at % concentration, probably due to its positioning in the graphene foam network, wither inside graphene matrix or located at the edges. A graphitic N component was present in all the nitrogen-doped samples, with a concentration varying from 15.7 at %, 7.8 at %, 13.1 at %, and 9.6 at % for GrFoam1N, GrFoam2N, GrFoam3N, and GrFoam1NPt, respectively, and a binding energy position situated between 404.85 eV and 401.87 eV. The pyridinic oxide N component was detected only in the case of GrFoam1NPt at 406.27 eV and with 11 at % concentration, and this was a result of the platinum anchoring and stabilization within the GrFoam1N support due to the presence of nitrogen. The obtained results are in good agreement with others reported in the literature [20,25]. Li et al. [20] reported that the nitrogen-doped graphene at a low temperature in the static air can be synthetized using thermal treatment of graphene oxide with urea (NG-Urea-air). They demonstrated that urea is the best precursor for synthesizing nitrogen-doped graphene with a relative high doping level (18.7 at %). The N 1s fitting of NG-Urea-air exhibits three main contributions: pyridinic N located at 398.5 eV (53.3 at %), pyrrolic N located at 399.8 eV (32.5 at %) and graphitic N located at 401.0 eV (12.2 at %). Deng et al. [25] synthesized nitrogen-doped graphene via the reaction of tetrachloromethane with lithium nitride under mild conditions. They indicated that the atomic ratio of N/C can be estimated from the peak areas of C 1s and N 1s. Fitting the N 1s signal indicates the presence of pyridinic N at 398.7 eV, pyrrolic N at 400.1 eV, and graphitic N at 401.8 eV. The XPS survey data obtained from the platinum interaction with the graphene foam matrix are presented in Figure 2e,f, and the content of Pt(4f) is shown in Table S3. According to these, in the case of GrFoamPt the Pt(4f) doublet binding energy was at 70.75 eV and 74.05 eV corresponding to the Pt being in the metallic forms Pt^0^(4f^7/2^) and Pt^0^(4f^5/2^) (Figure 2e). In the case of GrFoam1NPt, the Pt(4f^7/2^) and Pt(4f^5/2^) doublet was deconvoluted in the metallic state Pt^0^(4f^7/2^) and Pt^0^(4f^5/2^), platinum oxide (PtO) with the Pt^2+^(4f^7/2^) and Pt^2+^(4f^5/2^) states, and platinum oxide (PtO_2_) with the Pt^4+^(4f^7/2^) and Pt^4+^(4f^5/2^) components (Figure 2f) [42]. In conclusion, nitrogen shifts the binding energy position and forms platinum platinum-nitrogen links with the graphene foam matrix. This behavior influences the electrochemical properties with respect to HER.

### 5.2. Scanning Electron Microscopy (SEM)

**Figure 3:**
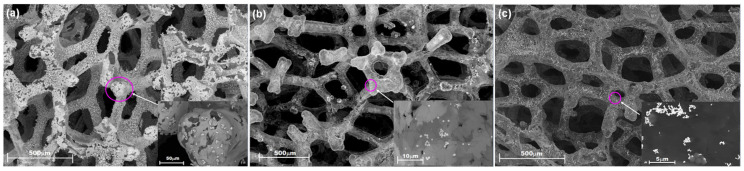
Top view and enlarged morphology of (**a**) pristine porous graphene after Ni scrapping; (**b**) graphene foam doped with nitrogen; and (**c**) platinum functionalized graphene foam.

As shown in Figure 3a–c, the morphology of porous carbon specimens after Ni removal consists of interwoven graphene layers and conglomerated carbon spherules with diameters between 0.6 and 1.3 μm, on top of a more uniform and compact graphene-like structure with wrinkles due to the dilatation of the nickel substrate during the high-temperature CVD process. Chemical scrapping of Ni results in exfoliated graphene with C-vacancies that can be occupied (doped) with nitrogen and platinum upon hydrothermal treatment with reducing agents (i.e., urea, hydrazine, ammonia, and dihydrogen hexachloroplatinate (IV) hydrate). However, after hydrothermal nitrogen doping, the GrFoam structure is preserved and stable, as is shown in Figure 3b. Moreover, the high magnification of the Figure 3c inset shows that platinum is anchored both on the graphene foam and on the carbon sphere surface. Therefore, the graphene foam structure can accommodate nitrogen and platinum functional groups for fuel cell and electrolysis applications.

As shown in Figure 3a–c, the morphology of carbon specimens is complex as a result of both the removal of Ni by chemical exfoliation in a strong acid and the N-doping by hydrothermal treatment with reducing agents in air. From these processes, conglomerated carbon spherules are formed on top of a more compact multi-layer graphene-like structure. Since the laser spot scans an irregular topography, information from both the carbon spherules and the graphene support are concomitantly obtained [16]. Similar observations were also made by Li et al. [20], who showed that N-doped graphene presents a curled structure. Nemiwal et al. [43] reported that the flexible and conductive graphene layer prevented cobalt phosphide nanosheet aggregation.

### 5.3. Raman Spectroscopy

**Figure 4:**
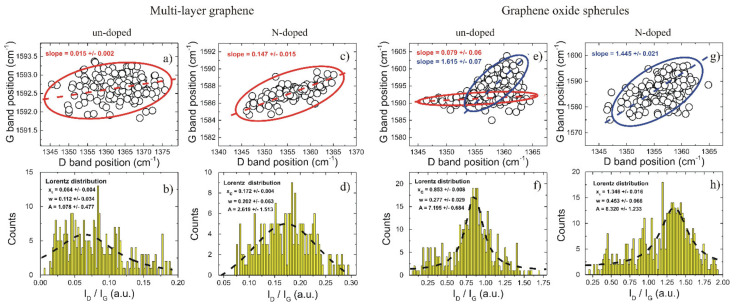
Raman analyses of multi-layer graphene (**a**–**d**) and graphene oxide spherules (**e**–**h**) from 25 × 25 µm^2^ scans with a 532 nm laser beam and a 20× magnification objective lens. The position of the G and D bands are shown for both carbons, as resulted from fit to data corresponding to un-doped and N-doped specimens. The data grouped in clusters, as indicated by each ellipse, whose main axis (i.e., indicated by dashed lines) slope denotes the correlation between the data. Additionally, the histograms associated with the intensity ratios of the D and G bands are given, respectively. The dashed lines represent fits to data using one Lorentz distribution. The parameters of the Lorentz distributions (i.e., x_c_—peak position, w—full width at half maximum (FWHM) and A-area) are indicated accordingly.

In Figure 4a, the dispersion of the G band with respect to the D band determined on un-doped specimens is very weak. The data clustered around 1592.5 cm^−1^, although the D band position was spread between 1345 and 1375 cm^−1^. The correlation between the data was given by the cluster main axis orientation, whose slope was 0.015 ± 0.002. These features are typically observed in strained multi-layer graphene (or bulk graphite). This is also supported by the I_D_/I_G_ ratio (in Figure 4b), with values below 0.20. A fit to histogram data using one Lorentz distribution yields x_c_ = 0.064 ± 0.004. This value is an indicator of highly ordered multi-layer graphene with a very small number of defects, which arise most probably from the domain edges. Compared to un-doped specimens, the N-doped specimens in Figure 4c,d present different features in both the dispersion of the G band position, with values between 1584 and 1589 cm^−1^, and the center of the I_D_/I_G_ distribution, which was shifted to higher values (i.e., x_c_ = 0.172 ± 0.004). Additionally, the cluster slope denoting the correlation between the G and D bands is nearly one order of magnitude higher (Figure 4c), with a slope = 0.147 ± 0.004, than that of un-doped specimens. The FWHM value of the Lorentz distribution in the histogram data (Figure 4d), with w = 0.202 ± 0.063, is almost double than that of un-doped samples (Figure 4a) with w = 0.112 ± 0.034. These features indicate that the hydrothermal treatment with reducing agents in air results in exfoliated graphene down to a few monolayers (i.e., with small G band position values of ~1582 cm^−1^). This offers the possibility for N doping from urea, either at domain edges (i.e., on pyrrolic and pyridinic positions) or inside graphene structures (i.e., on graphitic positions). The obtained results are in good correlation with those related regarding the morphology of porous carbon specimens after Ni removal, as well as graphene foam un-doped and doped with nitrogen. Li et al. [20] reported their synthesis of nitrogen-doped graphene via low-temperature pyrolysis. The Raman spectra for all samples (graphene oxide, nitrogen-doped graphene, and nitrogen-doped graphene in static air) exhibited two bands at 1583.2 cm^−1^ (G band) and 1331.8 cm^−1^ (D band). In the Raman spectra, carbon spherules exhibit D and G bands with nearly identical intensities, although a true de-convolution of the corresponding spectra of un-doped specimens indicate that the intensity ratio I_D_/I_G_ is preponderantly sub-unitary (Figure 4f), with x_c_ = 0.853 ± 0.008. This is commonly observed among graphene oxide specimens. For comparison, the histogram (Figure 4f) is far more complex than those obtained for multi-layer graphene. This is indicated by the very low I_D_/I_G_ values (i.e., with I_D_/I_G_ < 0.50) associated with multi-layer graphene flakes and the high values (i.e., with I_D_/I_G_ > 1.10) commonly observed in reduced graphene oxide. In Figure 4e, the dispersion of the G and D bands was bimodal; i.e., the red cluster with very low dispersion (i.e., slope = 0.076 ± 0.008) was comparable to that obtained for un-doped multi-layer graphene and the blue cluster with very high dispersion (i.e., slope = 1.615 ± 0.007) whose main weight is given by the Lorentz distribution (Figure 4f). However, hydrothermal treatment with reducing agents in air shifted the weight of the Lorentz distribution (Figure 4h) to x_c_ = 1.346 ± 0.018. This is evidence for the reduction reaction occurring on the carbonic spherules which may also provide defect sites for N-doping upon treatment with urea. Since samples with sub-unitary I_D_/I_G_ values are observed in Figure 4h, it is highly likely that the N-doping process was effective at the spherule surface rather than in bulk. In this framework, compacted spherules may form at the expense of adhesion surface energy. This is accompanied by modifications of the energy barrier at grain boundaries in a way that further N-diffusion in bulk carbon spherules is inhibited. Similar results were found by Li et al. [20], who reported that the ratio of D band/G band intensities (I_D_/I_G_) reflects the defective degree of the carbon materials.

### 5.4. FTIR Analysis

**Figure 5:**
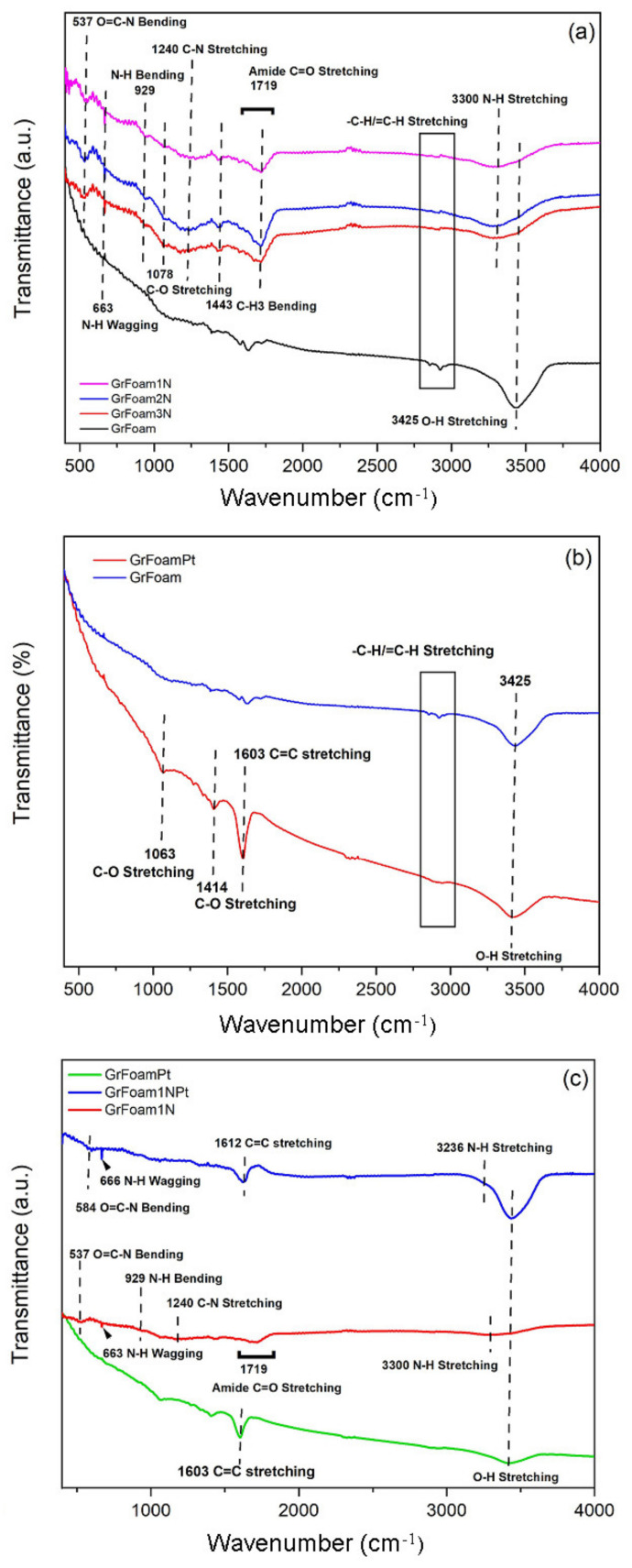
The overlaid FTIR spectra of: (**a**) GrFoam1N, GrFoam2N, and GrFoam3N; (**b**) GrFoamPt; (**c**) GrFoam1N and GrFoam1NPt, in comparison with pristine GrFoam.

Figure 5a–c presents the overlaid FTIR spectra of GrFoam1N, GrFoam2N, GrFoam3N, GrFoamPt, and GrFoam1NPt, in comparison with the pristine GrFoam sample. The large peaks from 3425 cm^−1^, 3300 cm^−1^, and between 2850–2925 cm^−1^ are associated with O-H, N-H, and -C-H/=C-H stretching vibrations, respectively. The C-O stretching vibration peak positioned at 1603 cm^−1^ in the GrFoam sample was shifted to 1612 cm^−1^ in the GrFoamPt sample, which is attributed to the Pt bonding with the porous graphene and carbon spherule substrate. The band from 1719 cm^−1^ ascribed to the amide C=O stretching vibration is seen in all the nitrogen-doped samples, and this is related with the COOH groups located at the graphene oxide margins [19,20]. The GrFoam-doped samples present several C-N and N-H vibration modes, i.e., C-N stretching, N-H bending, N-H wagging, and O=C-N bending, corresponding to 1240 cm^−1^, 929 cm^−1^, 663 cm^−1^, and 537 cm^−1^, respectively [14,41]. The N-H wagging and O=C-N bending bands were shifted to 666 cm^−1^ and 584 cm^−1^, respectively, for the GrFoam1NPt sample due to the Pt interaction with the GrFoam1N structure. The FTIR spectra suggested that nitrogen-containing groups were presented after the N-doping process, which is consistent with the XPS studies.

The structural, compositional and morphological characteristics of the samples suggests the efficient functionalization of using nitrogen and platinum anchored to the pristine GrFoam substrate.

## 6. Hydrogen Evolution Reaction (HER)

**Figure 6:**
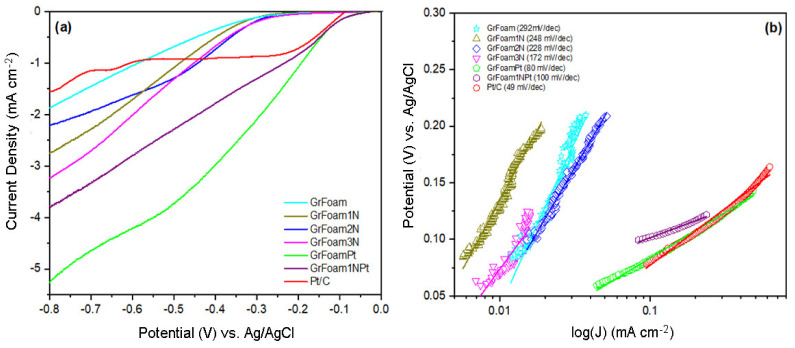
(**a**) Linear sweep voltammetry (LSV) plots recorded with a scan rate of 5 mV s^−1^ in nitrogen saturated 0.1 M KOH; and (**b**) potential vs. logarithm of current density in absolute values and the calculated Tafel slopes are obtained from linear fitting.

The catalytic activity towards hydrogen evolution reaction was analyzed from linear sweep voltammetry (LSV) plots recorded with a scan rate of 5 mV s^−1^ in nitrogen saturated 0.1 M KOH (Figure 6a), and corresponding Tafel slopes determined from Tafel Equation (6):

(6) $$\eta =a+blog\left(j\right),$$

where *η* is the overpotential that is the potential necessary to move the electrons at the electrode surface; *b* is the Tafel slope, and *j* is the current density.

Figure 6 shows the plots of the potential versus the logarithm of current density in absolute values, and the calculated Tafel plots obtained from linear fitting.

From Figure 6a, is observed that the current density increases with urea quantity. Although Pt/C, GrFoamPt, and GrFoam1NPt had similar onset, the Tafel slopes determined from the Tafel equation were 49 mV dec^−1^, 80 mV dec^−1^, and 100 mV dec^−1^, respectively (Figure 6b). Moreover, the associated Tafel slopes of GrFoam, GrFoam1N, GrFoam2N, and GrFoam3N were 292 mV dec^−1^, 248 mV dec^−1^, 228 mV dec^−1^, and 172 mV dec^−1^, respectively. The decreasing of the Tafel slope and the onset potential together with nitrogen amount enhancement demonstrates the positive effect that nitrogen doping has towards the HER of 3D-doped graphene foam. This is due to the presence of reduced sites and defects produced during nitrogen doping, and it is shown in Raman spectra that it influences the catalytic activity the samples. Comparing with an acid environment, the alkaline Tafel slopes are higher and the mechanism of HER is slower. In the case of all nitrogen-doped graphene foam samples, the Tafel plots values are higher than 120 mV dec^−1^, which means that the HER takes place through the Volmer–Heyrovsky mechanism, which involves water dissociation and proton adsorption (Volmer step) followed by electrochemical reduction and hydrogen molecule formation. For the platinum-functionalized graphene samples, the Tafel slope decreased, and the rate-limiting step for the HER is the Volmer–Tafel mechanism, in which two adsorbed hydrogens recombine and form a molecule of hydrogen. These mechanisms control electron transfer through the electrode and represent the rate-limiting steps for HER [44,45]. Zeng et al. [44] reported that HER is very sluggish in the alkaline media because water dissociation is required in the Volmer step. Li et al. [15] reported that the prepared nanostructures (free-standing N-enriched C foam@WS_2_ nanoflakes) have excellent electrocatalytic activity with a low overpotential of 153 mV at a current density of −10 mA cm⁻² and a small Tafel slope of 58.7 mV dec⁻¹.

## 7. Conclusions

In summary, this paper presents our results in approaching metal-free catalyst synthesis using the hydrothermal nitrogen-doping method of porous graphene obtained by the CVD method, in comparison with platinum trapping on the graphene support.

It was proven that it is possible to use graphene foam (GrFoam) for high corrosion resistance in alkaline environments, an enhanced mass transport (water and gases) flow field, and catalytic support. The replacement of porous metal in electrolysis systems (e.g., nickel, titanium), and the development of lightweight, economical, durable, and good electrochemical activity catalysts for HER are very important. In this paper, it was shown that, through hydrothermal treatment of GrFoam with different quantities of urea combined with ammonia and hydrazine at high temperature and under a nitrogen atmosphere, it was possible to produce nitrogen-doped graphene catalysts for HER. From the plot of the potential vs. the logarithm of current density in absolute values, it can be concluded that the most electrochemical active nitrogen-doped sample was GrFoam3N. This sample contains 1.8 at % of N, and it exhibits a 172 mV dec^−1^ Tafel plot associated with the Volmer–Heyrovsky hydrogen evolution (HER) mechanism in 0.1 M KOH.

Raman imaging data scans indicate that the D and G band positions are highly correlated, such that un-doped and N-doped specimens were distinct. Data analysis offers the possibility to discretize among multi-layer graphene and graphene oxide carbons, which are also self-organized in compacted spherules. The effect of N-doping on graphene foams is reflected in both the cluster relation between the D and G band positions and the I_D_/I_G_ histograms, whose Lorentz distributions shift to higher values upon hydrothermal treatment with reducing agents in air. As a result, N-doping is promoted on defective C-sites by the presence of exfoliated graphene and the high specific area of graphene oxide spherules. Moreover, platinum deposited by the hydrothermal method on both pristine and nitrogen-doped porous graphene is anchored to both the graphene foam and the carbon sphere surfaces. In conclusion, we may state that the GrFoam complex network produced by CVD can accommodate nitrogen and platinum functional groups for fuel cells and electrolysis applications.

## Figures and Tables

**Table 1:** Surface atomic composition for nitrogen and platinum functionalized graphene foam determined from XPS measurements.

Sample ID	Surface Atomic Composition (at %)			
	C	O	N	Pt
GrFoam	94.5	5.5	–	–
GrFoam1N	91.6	7.2	1.2	–
GrFoam2N	90.6	7.8	1.6	–
GrFoam3N	88.9	9.3	1.8	–
GrFoamPt	73.7	26.1	–	0.2
GrFoam1NPt	67.2	31.4	1.2	0.2

## Data Availability

Not applicable.

## Supplementary Materials

The following are available online at https://www.mdpi.com/article/10.3390/ma14174952/s1, Table S1: C1s peak position and content (at %) for every carbon element determined from XPS measurements, Table S2: N1s peak position and content (at %) for every nitrogen element determined from XPS measurements, Table S3: Pt4f peak position and content (at %) for every platinum element determined from XPS measurements.
