# Supplementary material for: Nitrogen Functionalization of CVD Grown Three-Dimensional Graphene Foam for Hydrogen Evolution Reactions in Alkaline Media
Source: Materials (Basel). 2021 Aug 30;14(17):4952. doi: 10.3390/ma14174952 (PMC8433733; doi:10.3390/ma14174952)
Supplement: Supplementary file 1 [file materials-14-04952-s001.zip › materials-1325727-supplementary.pdf]

materials

MDPI

Supplementary Materials

Nitrogen Functionalization of CVD Grown Three-Dimensional Graphene Foam for Hydrogen Evolution Reactions in Alkaline Media

Daniela Ion-Ebrașu [1], Radu Dorin Andrei [1], Stanică Enache [1], Simona Căprărescu [2,*], Constantin Cătălin Negrilă [3], Cătălin Jianu [1], Adrian Enache [1], Iulian Boerașu [4], Elena Carcadea [1], Mihai Varlam [1], Bogdan Ștefan Vasile [4] and Jianwei Ren [5]

[1] National Institute for Cryogenics and Isotopic Technologies ICSI-Rm. Valcea, ICSI Energy, Uzinei Street, no. 4, 240050 Ramnicu Valcea, Romania; daniela.ebrasu@icsi.ro (D.I.-E.); radu.andrei@icsi.ro (R.D.A.); stanica.enache@icsi.ro (S.E.); Catalin.jianu@isci.ro (C.J.); Adrian.enache@icsi.ro (A.E.); elena.carcadea@icsi.ro (E.C.); mihai.varlam@icsi.ro (M.V.)

[2] Inorganic Chemistry, Physical Chemistry and Electrochemistry Department, Faculty of Applied Chemistry and Materials Science, University POLITEHNICA of Bucharest, Gh. Polizu Street, no. 1–7, 011061 Bucharest, Romania

[3] National Institute for Materials Physics, P.O. Box MG-7, 77125 Bucharest, Romania; catalin.negrila@infim.ro

[4] National Research Center for Micro and Nanomaterials, Faculty of Applied Chemistry and Materials Science, University POLITEHNICA of Bucharest, Splaiul Independentei Street, no. 313, 060042 Bucharest, Romania; iulianboerasu@gmail.com (I.B.); bogdan.vasile@upb.ro (B.S.V.)

[5] Department of Mechanical Engineering Science, University of Johannesburg, Cnr Kingsway and University Roads, Auckland Park, Johannesburg 2092, South Africa; jren@uj.ac.za

* Correspondence: simona.caprarescu@upb.ro

Table S1. C1s peak position and content (at %) for every carbon element determined from XPS measurements.

C-O	C=O		O-C=O		Sp^2 hybridizatio n characteristi
Content (at %)	Position (eV)	Content (at %)	Position (eV)	Content (at %)	Position (eV)
5.1	286.05	4.2	-	-	-
6.6	286.23	4.1	-	-	-
4.7	286.61	2.7	-	-	-
3.9	286.77	1.5	-	-	-
13.2	286.95	11.2	288.76	13.6	290.41
15.9	286.81	14.7	288.39	18.4	290.22

Sample ID	C=C sp^2		C-C sp^3			
	Position (eV)	Content (at %)	Position (eV)	Content (at %)	Position (eV)
GrFoam	283.88	**86.7**	284.80	4	285.29
GrFoam1N	**283.90**	**85.5**	284.81	3.7	285.46
GrFoam2N	283.91	82.3	284.89	10.4	285.70
GrFoam3N	283.91	**84**	284.97	10.7	285.88
GrFoamPt	**284.13**	53.7	285.04	8.2	285.60
GrFoam1N Pt	**284.15**	**43.4**	285.10	7.6	285.62

Table S2. N1s peak position and content (at %) for every nitrogen element determined from XPS measurements.

Pyrrolic N		Graphitic N		Pyridinic oxide N	
Position (eV)	Content (at %)	Position (eV)	Content (at %)	Position (eV)	Content (at %)
-	-	404.85	15.7	-	-
399.69	51.2	402.03	7.8	-	-
400.12	52.4	401.87	13.1	-	-
399.51	40.5	402.39	9.6	406.27	11.00

Sample ID	Pyridinic N	
	Position (eV)	Content (at %)
GrFoam1N	399.74	**84.3**
GrFoam2N	398.30	41.4
GrFoam3N	399.18	**34.5**
GrFoam1NPt	**398.03**	**39.00**

Table S3. Pt4f peak position and content (at %) for every platinum element determined from XPS measurements.

$Pt^{2+}(4f^{7/2})$	$Pt^{4+}(4f^{7/2})$		$Pt^{0}(4f^{5/2})$		$Pt^{2+}(4f^{5/2})$		$Pt^{4+}(4f^{5/2})$	
Content (at %)	Position (eV)	Content (at %)	Position (eV)	Content (at %)	Position (eV)	Content (at %)	Position (eV)	Content (at %)
-	-	-	74.05	-	-	-	-	-
16.4	74.13	6.1	74.44	-	76.03	-	77.23	-

Sample ID	$Pt^0(4f^{7/2})$		
	Position (eV)	Content (at %)	Position (eV)
GrFoamPt	70.75	100	-
GrFoam1NPt	71.16	77.5	72.70
